# Supplementary material for: Coupling relationship between cold chain logistics and economic development: A investigation from China
Source: PLoS One. 2022 Feb 25;17(2):e0264561. doi: 10.1371/journal.pone.0264561 (PMC8880921; doi:10.1371/journal.pone.0264561)
Supplement: S1 File — (DOCX) [file pone.0264561.s002.docx]

# Conflict of interest

The authors declared that they have no conflicts of interest to this work. We declare that we do not have any commercial or associative interest that represents a conflict of interest in connection with the work submitted.
