# Supplementary material for: Coupling relationship between cold chain logistics and economic development: A investigation from China
Source: PLoS One. 2022 Feb 25;17(2):e0264561. doi: 10.1371/journal.pone.0264561 (PMC8880921; doi:10.1371/journal.pone.0264561)
Supplement: Attachment — Submitted filename: Review 1-Answer.docx [file pone.0264561.s003.docx]

**Review 1**

Dear professor,

Thank you for your comments. Your suggestions have helped me a lot; we have made a revision of the paper again. The language and grammar of the paper have been rechecked by the professional polishing institutions. The answers to the expert questions are as follows. For details, you can view the revised version.

1.Highlight the innovation and value of the paper. Add scientific value or novelty in the introduction. There are some editing/grammar errors throughout the paper that need to be corrected. This paper needs a round of professional proofreading.

Answer:

1. **Introduction**

As the rapid development of the global economy intensifies the process of urbanization, and consumer requirements and expectations regarding the quantity and quality of fresh food continue to rise, cold chain logistics becomes the fundamental prerequisite to fulfilling consumer requirements [1]. The term refers to a systematic process that ensures that refrigerated and frozen food is always stored and transported in an appropriately low-temperature environment at all stages, from production to consumption, to guarantee quality and reduce waste and loss to a minimum [2-4]. According to the report of the China Cold-chain Logistics Association [5], the market demand for cold chain logistics in China increased by 17.5% in 2019, and the market size is expected to reach RMB 915 billion (US $140 billion) in 2025, with an average annual growth rate of 18% that should provide tremendous potential for the overall increase of the market economy.

It is worth noting that the relationship between cold chain logistics and economic development is becoming increasingly close [6]. The development of the former drives the latter by allowing macro-economic control, innovating diversified economic development modes, stabilizing prices and farmer income, and guaranteeing food safety [1, 7-9]. In turn, creating and supporting a solid industrial infrastructure, providing ample financial support, stimulating market demand potential, and enhancing industry service professionalization can spur the development of cold chain logistics [10-12]. However, if the overall economic development cannot generate sufficient demand for cold chain logistics capacity, further investment in human, financial, and other resources will only result in a waste of governmental and social resources. Reversely, if the input of government and social resources fails to meet the developmental needs of cold chain logistics, the consequences on the safety of the transported fresh agricultural products and the quality of life of consumers will undermine the overall economic development and the vitality of the sector in particular, as neither will be able to reach its full potential [10]. In other words, cold chain logistics and the economy are very likely to benefit from mutual, combined, coordinated growth, which is the optimal option to maximize the profits of stakeholders. However, current studies mainly focus on the impact of cold chain logistics on the economy, tend to neglect the reverse influence, and fail to further enquire on their coordinated development. Therefore, our research makes important contributions to our understanding of their interaction.

This study analyzes the interaction between Chinese cold chain logistics and the economy. Nowadays, with the help of information technology (e.g., blockchain, Internet of Things, big data, cloud computing, Al), cold chain logistics is in a better position to assist suppliers in managing orders, controlling inventory, meeting consumer demand for convenience and timely delivery, and benefit from economies of scale [6, 13, 14]. In terms of benefits generation, economic growth encourages the formation of innovative processes for cold chain logistics to optimize the supply chain, which increases consumer demand for products reliant on cold chain logistics and stimulate further development of the industry.

Therefore, this study revolves around the correlation between cold chain logistics and economic development and examines their coupling relationship. It is important to capture the coordination mechanism among various factors of cold chain logistics and economic development theoretically and empirically and to explore effective methods to measure the two.

This paper aims to build an evaluation framework with an index system that can evaluate the coordinated development level of cold chain logistics and economy, in order to analyze and measure the coupling relationship between cold chain logistics and economic development, to determine the main factors affecting their coordinated development, and to suggest specific measures to enhance their coordination. The paper is structured as follows. Section 2 outlines trends in existing literature on the interaction between cold chain logistics and the economy. Section 3 constructs the index evaluation system and presents the research methods, namely EWM, CCMD, and GRA. Section 4 analyzes the coordinated development of cold chain logistics and the economy. Section 5 proceeds with an analysis and discussion of the influencing factors affecting their coordinated development, and outlines policy recommendations, Section 6 concludes the paper and Section 7 briefly expounds on research limitations and suggests directions for future research.

2. **Introduction**

In an era marked with enormous changes in consumption habits and patterns, scholarly studies focus on the sustainable development of logistics and the economy, but without elaborating specifically on the coordinated development relationship between cold chain logistics and the economy. The metropolitan economy contributes to the increase in demand for logistics and the investment in infrastructure, thus enhancing the coordinated development of the metropolitan economy and logistics [15, 16]. More significantly, there is a positive interaction between the metropolitan economy and logistics [10]. The global economy and improved living standards have diversified the demand for logistics. The development of cold chain meets consumer demand by ensuring timely delivery of a wide range of products, from fresh food delivery to vaccines shipment. Cold chain is an extremely important sector of the logistics industry and has made a substantial contribution to a developing Chinese economy in recent years [1].

Regarding the cold chain logistics operation mode, Liu et al. [17] created a Joint Distribution-Green Vehicle Routing Problem model and concluded that joint distribution in cold chain logistics can balance economic and environmental benefits. Rodrigues et al. [18] analyzed the factors affecting the efficiency of third-party logistics suppliers of refrigeration services in Brazil and made suggestions to tackle challenges in the supply chain to improve company performance. Some scholars considered the relevant technologies to optimize the operation of cold chain logistics. Defraeye et al. [19] suggested ways to optimize the pre-cooling technology for fresh agricultural products before they are placed into refrigerated containers and decrease the heat load in the cold chain logistics chain to reduce energy consumption. Dao et al. [20] studied the insulation materials used for vaccine transport containers and found that the basic algorithm developed to simulate complex heat transfer processes can also be a fast and economical tool for screening suitable phase change materials to reduce transportation costs. Li et al. [21] applied the Internet of things technology to real-time monitoring of safety and quality parameters of perishable products, such as temperature and humidity during transportation, in the cold chain logistics transportation process. Other researchers probed into the influencing factors of cold chain logistics. Zhang et al. [22] discussed the impact of government subsidies towards reducing carbon emissions and relevant trading policies on regional cold chain logistics, and provided suggestions for the government and enterprises to support their joint effort to form a low-carbon economy and an energy-conservation mentality. In times of global economic growth, Korean logistics enterprises chose cold chain logistics with higher added value to enter overseas markets, and pointed out that the development of an emerging logistics market cannot do without the cooperation between central and local governments and private enterprises, as well active strategies to encourage investment [23, 24]. In addition, social, environmental, and technological factors also influence the sustainable development of cold chain logistics [6, 21].

However, those studies seldom explore the influencing factors that indicate the development level of cold chain logistics, which are important in policymaking and innovating operation modes. In addition, the role of the economy in promoting cold chain logistics development is not fully considered.

Meanwhile, the influence of economic development on cold chain logistics has attracted significant scholarly attention. Qin and Tian [25] used the grey clustering method to analyze the development level of cold chain logistics in regions with significant agricultural production in China. The results revealed that regional differences in economic development and resource distribution affect the regional development level of Chinese agricultural cold chain logistics. The higher the pace of economic progress and the richer the resources, the higher the level of regional agricultural cold chain logistics development. Park [26] investigated the influencing factors that determine the location of global cold chain logistics hubs by employing the analytic hierarchy process (AHP) method, which revealed that the market appeal of the sector was the primary factor. Ikegawa and Tokunaga [27] studied the factors influencing the location of Japanese frozen food companies in foreign countries. They found that the geographical factors that determine a company’s decision to enter foreign markets are affected by the popularity of refrigerators and by well-known cost reduction factors, such as wage levels, levels of national investment, and support from government policies. Zhang and Pang [28] estimated that the cold chain logistics joint distribution technology in developed countries is generally mature, and the level of urban economic development affects the development of urban fresh agricultural products cold chain logistics. That is, the more prosperous the urban economy, the swifter the development of urban fresh agricultural products cold chain logistics, and their research mainly focuses on the cold chain logistics for agricultural products. Also, economic growth has boosted the consumers’ disposable income and improved living standards. Consequently, consumers attached great importance to timely delivery, safety and quality of fresh agricultural products, and scholars are exploring ways to create and optimize cold chain logistics processes in terms of vehicles [6, 29], inventory strategy [30, 31] and information transmission [32, 33], In turn, all these contribute to the development of cold chain logistics.

Despite the extensive scholarly attention and thorough research on the topic, existing literature neglects several important aspects, which can enhance our understanding of the close relationship between cold chain logistics and the economy. First, in the empirical analyses of the relationship between cold chain logistics and economic development, existing research lacks a comprehensive evaluation of the development level of cold chain logistics. Current research only aims at probing the service level or benefits level of cold chain logistics and, to the best of our knowledge, not a single study attempts to appreciate the overall level of development of cold chain logistics. Second, only a few scholars have investigated the development of cold chain logistics and the economy in China, and none has approached it from a macro perspective. Third, scholars have concentrated their attention on the impact of cold chain logistics on the economy and fail to fully appreciate the correlation between economic factors and cold chain logistics, such as GDP per capita, per capita disposable income gap, and household consumption level. Instead, scholars dwell more on the indirect impact of economic development on cold chain logistics. Therefore, it is necessary to construct a reliable and applicable evaluation system to measure the coupling coordinated relationship of cold chain logistics and economic development, to analyze the main factors affecting their coordinated development, to provide useful insights, and to suggest future directions for the development of the industry and the economy.
